# Penfield’s Prediction: A Mechanism for Deep Brain Stimulation

**DOI:** 10.3389/fneur.2014.00213

**Published:** 2014-10-20

**Authors:** Richard W. Murrow

**Affiliations:** ^1^Department of Neurology, University of North Carolina, Chapel Hill, NC, USA; ^2^Department of Neurosurgery, University of North Carolina, Chapel Hill, NC, USA

**Keywords:** deep brain stimulation, parallel processing, forebrain, neural synchronization, Penfield, DBS mechanism of action, history of DBS, thalamus

## Abstract

**Context:** Despite its widespread use, the precise mechanism of action of Deep Brain Stimulation (DBS) therapy remains unknown. The modern urgency to publish more and new data can obscure previously learned lessons by the giants who have preceded us and whose shoulders we now stand upon. Wilder Penfield extensively studied the effects of artificial electrical brain stimulation and his comments on the subject are still very relevant today. In particular, he noted two very different (and seemingly opposite) effects of stimulation within the human brain. In some structures, artificial electrical stimulation has an effect, which mimics ablation, while, in other structures, it produces a stimulatory effect on that tissue.

**Hypothesis:** The hypothesis of this paper is fourfold. First, it proposes that some neural circuits are widely *synchronized* with other neural circuits, while some neural circuits are *unsynchronized* and operate independently. Second, it proposes that artificial high-frequency electrical stimulation of a synchronized neural circuit results in an *ablative* effect, but artificial high-frequency electrical stimulation of an unsynchronized neural circuit results in a *stimulatory* effect. Third, it suggests a part of the mechanism by which large-scale physiologic synchronization of widely distributed independently processed information streams may occur. This may be the neural mechanism underlying Penfield’s “centrencephalic system,” which he emphasized so many years ago. Fourth, it outlines the specific anatomic distribution of this physiologic synchronization, which Penfield has already clearly delineated as the distribution of his centrencephalic system.

**Evidence:** This paper draws on a brief overview of previous theory regarding the mechanism of action of DBS and on historical, as well as widely known modern clinical data regarding the observed effects of stimulation delivered to various targets within the brain. Basic science investigations, which support the hypothesis are also cited.

**Conclusion:** This paper proposes a novel hypothesis for the mechanism of action of DBS, which was conceptually foreshadowed by Wilder Penfield decades ago.

## Introduction

Artificial stimulation of the nervous system is not a new undertaking. Luigi Galvani noted in 1791 the stimulatory effect of electricity in animal tissue (1). In the first part of the nineteenth Century, Luigi Rolando (2) and Pierre Flourens (3) pioneered the use of electrical stimulation to study the localization of animal brain function. Contrary to prevailing opinion at the time, Eduard Hitzig and Gustav Fritsch demonstrated in 1870 that even certain portions of the surface of the animal brain produced a response to electrical stimulation (4). Artificial electrical stimulation in humans soon followed by Robert Bartholow (5) in 1874, Victor Horsley (6) in 1884, Charles Sherrington (7) in 1893, and Harvey Cushing (8) in 1909.

Several others followed and made important contributions, but the most detailed studies of the effect of artificial electrical stimulation on the human brain were made by Wilder Penfield and his colleagues over several decades of neurosurgical practice during the mid-portion of the Twentieth Century (9). Initially, artificial electrical stimulation was used primarily for localizing and diagnostic purposes. Later in the Twentieth Century, however, artificial brain stimulation began to be used for therapeutic purposes. Widespread use of therapeutic brain stimulation, however, did not begin until the introduction of high-frequency deep brain stimulation (DBS).

Deep brain stimulation is currently a widely used and effective therapy for a number of neurologic disorders. It is used most commonly to treat idiopathic Parkinson’s disease (PD) and severe essential tremor syndrome (ET). When it was first introduced in 1987, it was introduced as an alternative to ablative lesioning (10). The effect of DBS at its most common targets of the subthalamic nucleus (STN), ventral intermediate nucleus of the thalamus (VIM), and globus pallidus interna (Gpi) very much mimic the effect of small ablative lesions at these locations. One of the big advantages of DBS is the ability to modify the location and size of the “lesion effect” via post-operative programing of the implanted generator. This enables the physician to “fine-tune” the effects of stimulation to maximize the beneficial effects and minimize any stimulation-related adverse effects.

This paper has four sections. Section “Background to DBS” provides necessary background information, including a discussion of previous proposals for the mechanism of DBS, as well as a review of a paradox in common clinical experience with DBS, specifically that electrical stimulation through the DBS electrode sometimes produces an effect, which mimics ablation and other times produces a stimulatory effect. Section “Parallel Processing and Synchronization in the Brain” points out the importance of temporal synchronization in the parallel processing, which must occur in the forebrain. Section “Synchronized Parallel Forebrain Hypothesis” suggests a part of the mechanism for that synchronization and elaborates a hypothesis regarding the anatomical distribution of forebrain synchronization, which also explains the commonly observed paradox of the seemingly dual and opposite effects of DBS. Section “Penfield’s Prediction”, the final section, discusses how Penfield anticipated this fundamental conceptualization decades ago.

## Background to DBS

### Mechanisms of DBS: prior proposals

When DBS was first introduced, it was thought to produce its beneficial effects by producing a “depolarization block” of the neurons close enough to be affected by the artificially induced rapidly fluctuating electromagnetic field produced by the electrode (11). Neuron cell bodies were known to have a maximum firing rate. Stimulation of neurons above their maximum firing rate produces a continuous state of depolarization known as depolarization block during which subsequent firing of action potentials is not physiologically possible. This was thought to nicely explain why DBS effects so closely mimicked ablative effects.

It was soon realized, however, that this could not be the explanation. Even though the soma of the neuron may be in a depolarization block, the axons which emanate from the cell bodies and project out of the artificially induced voltage gradient field are capable of firing at and do fire at these fast “supra-physiologic” frequencies. In mammals, the STN neurons utilize glutamate in an excitatory synapse with its target neurons (the globus pallidus and substantia nigra pars reticulata). Microdialysis studies in the globus pallidus of the rat have shown increased levels of glutamate during STN stimulation, suggesting an activation of glutamatergic output from the STN to the globus pallidus (12). Likewise, recordings from the Gpi during DBS in non-human Parkinsonian primates have demonstrated increased mean discharge rates of those Gpi neurons (13). The question then arose, why does stimulation of some neural structures mimic the effect of ablation of those same neural structures?

An elegant explanation was then proposed by Grill et al., that the pathological “information content” of the signal between the STN and Gpi was being removed by the high-frequency stimulation (14). Because the information content of the signal was lost, it seemed to make sense that the effect should mimic ablation.

The question of the mechanism of action of DBS, however, has not been settled, and continues to be hotly debated and studied. A wide array of modern techniques has been applied to the problem. Examples include computational modeling (15), *in vitro* slice neurophysiology (16), *in vivo* microelectrode neurophysiologic studies (16), functional magnetic resonance imaging (17), positron emission tomography (18), quantitative microdialysis (12), and optogenetic neural circuit mapping (19). A variety of potential mechanisms of action of DBS, some of which are antithetical to one another, have been proposed. Examples include neuronal inhibition (20), orthodromic neuronal stimulation (13), antidromic neural stimulation (19), activation of adjacent fiber tracts (21), regularization of neuronal activity (22), elimination of pathologic oscillations (i.e., in the beta band) (23), inhibitory neurotransmitter release from afferent synaptic terminals (24), synaptic depression via neurotransmitter depletion (25), synaptic dopamine facilitation (26), reduction in pathologic information transmission (14), enhanced physiologic information transmission (21), and a combination of both reduction of pathologic activity and imposition of a beneficial frequency band (27).

### Ablative and stimulatory effects observed in DBS

While it is generally agreed that the beneficial effects of DBS at the usual targets somehow mimic the effect of ablation, it has been, nonetheless, commonly observed by clinicians in the field, that DBS many times, quite to the contrary, produces an actual *stimulatory* effect of neural tissue, and does *not* mimic ablation (28). An example can be seen when attempting to stimulate the STN or VIM. If the electrode is misplaced laterally, or, if the intensity of the stimulation is too high, then one may see activation of the corticospinal and corticobulbar tracts with resultant tetanic muscle contraction of the extremities and face. If DBS were mimicking a lesion at this site, then the expected outcome would be *paresis*, not muscle contraction. I have cataloged in Table 1, many sites which produce a direct and obvious physiologically defined ablative effect during DBS. In Table 2, I have cataloged, by contrast, many sites which produce an obvious physiologically defined stimulatory effect with DBS. In the past, it has been suggested that this differential effect was due to the difference between stimulating fiber tracts (white matter) and cell clusters (gray matter) (28). This, however, clearly cannot be the case, as the examples in the tables illustrate ablative effects sometimes in white matter and stimulatory effects sometimes in gray matter.

**Table 1:** **Ablative effect of high-frequency stimulation**.

**1.**	**VIM:** Part of the motor thalamus-projects to the motor cortex. Result of stimulation: loss of tremor
**2.**	**STN:** Neurons project to Gpi and substantia nigra pars reticulata as part of basal ganglia processing of sensory and motor information. Result of stimulation: loss of tremor, rigidity, and bradykinesia in patients with PD
**3.**	**GPi:** Neurons projecting to motor thalamus. Result of stimulation in PD: loss of tremor, rigidity, and bradykinesia. Result of stimulation in dystonia: delayed loss of dystonia
**4.**	**Ventral pallidum^a^:** Neurons projecting to dorsal medial nucleus of the thalamus. Result of stimulation: loss of depression and obsessive thinking
**5.**	**Language centers in the left cerebral cortex: Result of stimulation:** loss of normal language processing (aphasia)
**6.**	**Anterior limb of the internal capsule:** Axons projecting from the thalamus to the frontal lobes. Result of stimulation: loss of obsessive thinking

*^a^The target for obsessive–compulsive disorder has been described in the literature under many names including the nucleus accumbens and the ventral anterior limb of the internal capsule and adjacent ventral striatum (“VC/VS”). Over time, however, the target has moved posteriorly. In my opinion, the target is more accurately described anatomically as the ventral pallidum. Regardless of the name, the point made by inclusion of this target in this table is the same*.

**Table 2:** **Stimulatory effect of high-frequency stimulation**.

**1.**	**Central caudal nucleus of the thalamus:** Neurons relaying external tactile information to primary sensory cortices. Result of stimulation: persistent paresthesias
**2.**	**Posterior limb of the internal capsule:** Axons from the primary motor cortex descending in the corticobulbar and corticospinal tracts to lower motor neurons in the brainstem and cord. Result of stimulation: tetanic muscle contraction and spastic dysarthria
**3.**	**Optic tract:** Axons from the eye to lateral geniculate body. Result of stimulation: visual flashing
**4.**	**Medial hypothalamus to midbrain tectum:** result of stimulation: “flight or fight” response. OR: contralateral diaphoresis response
**5.**	**Supranuclear oculomotor fibers:** Fibers projecting from the frontal eye fields to the superior colliculus. Result of stimulation: involuntary conjugate contralateral eye deviation
**6.**	**Neocortex:** Direct electrical stimulation of the cortex usually produces an ablative effect. However, it is well known that *sometimes* direct cortical artificial electrical stimulation provokes a stimulatory effect. Result of stimulation over the primary motor cortex, for example, can invoke movements (see discussion in text)

There is also abundant evidence, in the historical literature, that artificial electrical brain stimulation sometimes produces a stimulatory effect and, at other times, produces an ablative effect. Penfield, in fact, described two principal effects of artificial electrical stimulation: “The first effect is electrical interference. The second is electrical activation” (9).

Why should there be two seemingly opposite effects of brain stimulation, and what implications does this have for the mechanism of action of DBS? I suggest that the answer to this paradox lies in dual forms of processing in the respective areas of the brain that are being stimulated. This will be elaborated upon in Section “Synchronized Parallel Forebrain Hypothesis”.

## Parallel Processing and Synchronization in the Brain

The reason there are two very different effects to brain stimulation is related to a fundamental concept, based on the obvious, but often ignored, observation that our brains are *parallel* processors, not just serial processors. Serial processing is the sort of processing that goes on in our recently humanly engineered computers. Human-made computers process one bit of information at a time, but do so at ever increasing speed, so that a large amount of information can be processed in a short amount of time. In serial processors, it is critical that the information be processed in the correct sequential order, that the information be “timed” correctly. This requires a mechanism for the correct timing or ordering of computations. In computers, this is accomplished by a “timing signal.” In parallel processing, however, by definition, multiple independent information streams are being processed simultaneously. In order to accomplish this, there must be some means to later combine those information streams to build higher order constructions. If multiple independently processed information streams are ultimately combined, it would be critical that they have the correct *temporal synchronization*. The neural mechanism, which must somehow accomplish this, I will refer to as, the “*synchronization mechanism*.”

There are many obvious examples that our brains routinely process information in parallel. Our visual association cortices combine independent aspects of visual information (e.g., edges, shape, and color) to later bring together recognition of a visual object (such as a hairbrush). Visual information is then later combined with independently processed auditory information to form a more unified “concept” (or distributed neural representation) of, for example, an individual person or event.

Though the focus of this paper is on physiologic synchronization in the forebrain, this is not to say that some forms of synchronization do not occur outside of the forebrain, or in a *pathologic* fashion. Certainly, epilepsy is an example of pathologic neural synchronization. Likewise, *physiologic* synchronization is a fundamental aspect of normal cerebellar processing (29). This circuitry is thought to provide the cerebellum the ability to compare the expected feedback (via pontocerebellar connections) with actual feedback (via spinocerebellar connections). This is likely important in the correction and timing of movements.

Forebrain physiologic synchronization, however, is something entirely different from the physiologic synchronization in the brainstem and cerebellum. Parallel synchronized processing in the forebrain occurs on a much larger scale and involves the merger of a very large number of independent information streams into very sophisticated constructions. There are likely multiple nested synchronized sub-circuits. These circuits likely combine to form still larger networked functional circuits. The largest of these combined circuits likely provide us with our most global concepts, such as the internal representation (or sense) of self, and thereby the recognition of the *meaning* of a perceived object within our environment. Meaning, by linguistic definition, implies a reference: “meaningful to whom?” A sense of self, therefore, is also necessary to produce meaningful movement (or behavior). The construction of an internal representation of the self as a special object within our environment is accomplished by the reprocessing of internal data in parallel and simultaneous with the processing of information about the environment. The forebrain provides for higher vertebrates the neural apparatus necessary for this special sort of parallel processing, processing that is capable of producing the rich repertoire of complex behaviors in response to ever changing environmental situations detected by our rich array of sensory systems.

## Synchronized Parallel Forebrain Hypothesis

### Requirements for a forebrain synchronization mechanism

Any temporal synchronization mechanism would require at a minimum the ability to provide a common “temporal tag” to multiple independent streams of information. If independent information streams are to be later merged, it would be essential that they be merged in the same moment in time. I propose that the common temporal tag must be supplied by an *instantaneous–simultaneous widely distributed* neural signal. It has been long known that the brain does produce many such signals, and that most emanate from the basal forebrain, for example, the *thalamus*. I suggest that it is misleading to think of the thalamus as simply a “relay.” It certainly does relay information to the striatum and cortex, but *processing* also occurs here. Part of that “processing” is the thalamic contribution to the eventual synchronization of independent parallel neural circuits in the forebrain (30).

The notion of the importance of temporal synchronization in neural circuits is not new. It was persuasively demonstrated by Wolf Singer in 1988 (31). He provided experimental evidence to support the presence of “temporal binding” in the processing of information in the visual cortex of the cat. The synchronization he referred to, however, involved *local cortically driven synchrony* necessary for the independent processing of specific visual information. It is not hard to imagine, however, that this processed information will later be merged (synchronized) with other information streams to produce larger and more complex neural constructs.

It was Donald Hebb, in the 1940s, who first proposed the concept of the “cell assembly,” an anatomically dispersed but functionally integrated ensemble of neurons, which act as a single functional unit via coordinated network activity (32). Later, the idea arose, that neural *synchronization* may be the neural mechanism that functionally binds the neurons together. Charles Gray, in 1994, highlighted the importance of synchronized oscillations in integrating anatomically disparate neural circuits (33). He writes: “Perhaps … synchronous rhythms have evolved to dynamically control the grouping of populations of cells organized in assemblies … [which] are essential for the coordination and integration of functions that are anatomically distributed.” (33). Others have highlighted the role the *thalamus* may play in coordinating and synchronizing activity from different cortical areas (34), and likely the cortico-basal ganglia–thalamocortical networks as well (30).

### Summary and the anatomical specifics of the synchronized parallel forebrain hypothesis

I would like now to return to the question discussed in Section “Ablative and stimulatory effects observed in DBS” regarding why DBS sometimes appears to “stimulate” neural tissue and at other times to “functionally ablate” neural tissue. I hypothesize, if stimulation occurs in a circuit that is *synchronized*, then the action potentials produced will be out of phase (out of synch) with the rest of the neural circuit and will therefore be “interpreted” only as noise. That is, the effect would be the same as simply removing the signal, or creating an “informational lesion” as has previously been proposed (14). If, however, the stimulation occurs in a *non-synchronized* circuit, then the effect of the stimulus will be propagated to the receiving neuron or end organ. In the case of corticospinal tract stimulation, the stimulus is propagated all the way to skeletal muscle.

The hypothesis can be summarized as follows (see Table 3): the hypothesis assumes the forebrain is organized as a single functional unit. The three principal components of the forebrain are the cortex, basal ganglia, and diencephalon (thalamus). The connection and communication between these three structures is so extensive, that it is misleading to consider the operation of one of these structures independent of the others. This functional unit does what the nervous system in general does; it provides a sensory-motor interface for the organism. The basal ganglia may be involved in selecting and enabling programed (learned) motor responses that do not need to be directed by conscious attention, freeing the cortex for other cognitive tasks. In any case, the elaboration of the forebrain is the principal change in nervous system evolution of higher vertebrates (amniotic vertebrates) (35). The development of the forebrain provides the neural apparatus necessary for the processing of independent information streams in parallel. Parallel processing, in turn, provides the sophisticated processing necessary for meaningful motor responses (i.e., behavior). There certainly is some parallel processing in non-forebrain structures such as the cerebellum, but this occurs on a much smaller and limited scale. In addition, there are obviously many separate brainstem circuits, which operate independently, but they, by and large, do not require synchronization and simultaneous merging with other brainstem circuits.

**Table 3:** **Synchronized parallel forebrain hypothesis**.

**I.**	**The forebrain functional organization**
A.	The three principal components of the forebrain – the cortex, basal ganglia, and diencephalon (thalamus) – operate as a single functional unit, which provides a sensory-motor interface for the organism
B.	The elaboration of the forebrain (cortex-basal ganglia–thalamus) is the principal change in evolutionary development of the higher vertebrates
C.	The development of the forebrain provides the neural apparatus necessary for the processing of independent information streams in parallel
D.	Parallel processing provides the increasingly sophisticated processing necessary for meaningful motor responses (i.e., behavior)
**II.**	**The synchronization mechanism**
A.	There must exist some mechanism by which these independent information streams are later merged to form higher order constructions
B.	That mechanism would require temporal synchronization of the independent information streams. This is referred to as the “synchronization mechanism.”
C.	Any synchronization mechanism would require the ability to provide a common “temporal tag” to multiple information streams
D.	That common temporal tag (the synchronizing signal) must be supplied by an instantaneous-simultaneous widely distributed neural signal
E.	The basal forebrain (thalamus) provides this signal
**III.**	**Synchronized and unsynchronized neural circuits**
A.	Most forebrain neural circuits are synchronized via the thalamic temporal tag signal
B.	Two classes of forebrain neural circuits, however, are unsynchronized: primary sensors and final effectors
C.	Artificial high-frequency electrical stimulation of synchronized neural circuits results in an ablative (“interference”) effect while artificial high-frequency electrical stimulation of unsynchronized neural circuits results in a stimulatory (“activation”) effect

The hypothesis asserts that there must be some mechanism by which independent information streams are later merged to form higher order constructions. That mechanism would require temporal synchronization. Any synchronization mechanism would require the ability to provide a common “temporal tag” to multiple information streams, and that tag must be supplied by an instantaneous–simultaneous widely distributed neural signal. The hypothesis asserts that it is the basal forebrain (primarily the thalamus), which provides this synchronizing signal.

Finally, the hypothesis suggests that most forebrain neural circuits are synchronized by the thalamic temporal tag, but that two classes of unsynchronized, independently operating forebrain circuits exist. Unsynchronized circuits occur in “*primary sensors*” and “*final effectors*.” “Final effectors” include neurons, which project to the “motor system broadly defined” by Swanson (36). That is, neurons which project to other neurons or effectors to produce an end behavior or response. This would include, for example, the deep layer V neurons of the primary motor cortex and premotor cortex, which project down to effectors in the brainstem and spinal cord. It would also include neurons in the medial hypothalamus, which project down to the midbrain locomotor region in the caudal tectum. It would also include final effectors in the autonomic and neuroendocrine systems. “Primary sensors” would include, for example, neurons and pathways from the primary sensory organ up to the receiving cells in layer IV of the primary sensory cortices.

### Evidence to support the synchronized parallel forebrain hypothesis

There is basic science work from non-human primates that “neuronal oscillations enable selective and dynamic control of distributed functional cell assemblies” (37). Specifically, spike timing in single neurons has been found to depend upon “oscillatory phase coupling,” which serves to “synchronize anatomically dispersed neuronal ensembles” (37). This is “thought to be responsible for computation and communication in large-scale brain networks” (37). Edward Jones has suggested that a “coincidence detecting circuit” is important in thalamocortical synchrony, which ultimately leads to the “act of perception” by “‘binding’ all the elements of an experience into a single cognitive event” (30).

The model in Table 3 would also explain the widely known clinical DBS observations made in Tables 1 and 2. *Ablative effects* of artificial high-frequency electrical stimulation are known to occur in the VIM, STN, GPi, Ventral Pallidum, and anterior limb of the internal capsule (ALIC). These are all circuits, which the model would expect to be *synchronized*. Therefore, stimulation of these structures would be expected to produce *ablative* effects. VIM stimulation in essential tremor results in loss of tremor. STN stimulation in PD results in loss of tremor, rigidity, and bradykinesia. GPi stimulation in PD also results in loss of tremor, rigidity, and bradykinesia. Stimulation of GPi in dystonia results in delayed loss of dystonia. Stimulation of the Ventral Pallidum results in loss of depression and obsessive thinking. Stimulation of the ALIC results in loss of obsessive thinking (38, 39). Another example, from common neurosurgical mapping experience, is seen with direct stimulation of the language cortices of the left hemisphere. Stimulation there results in aphasia (loss of language processing).

On the other hand, *stimulatory effects* of artificial high-frequency electrical stimulation are known to occur in the ventral caudal nucleus of the thalamus (Vc), the posterior limb of the internal capsule (PLIC), the optic tract (OT), the medial hypothalamus, and supranuclear oculomotor fibers. These are all circuits, which the model would expect to be *unsynchronized*. Therefore, stimulation of these structures would be expected to produce *stimulatory* effects. Stimulation of Vc produces paresthesias, not hypesthesia. Stimulation of the PLIC produces tetanic muscle contraction (not paresis) and spastic dysarthria. Stimulation of the OT results in visual flashing, not visual loss. Stimulation involving the medial hypothalamus results in a “flight or fight” response. Contralateral diaphoresis can be seen after stimulation in the area. Stimulation of supranuclear oculomotor fibers projecting from the frontal eye fields, and descending in the internal capsule, result in involuntary conjugate contralateral eye deviation. This is routinely seen during supra-therapeutic voltage test stimulation of DBS of the STN. Direct electrical stimulation of the cortex usually produces an ablative effect. However, it is well known that direct cortical artificial electrical stimulation on occasion provokes a stimulatory effect (5). Result of stimulation over the primary motor cortex, for example, can invoke movements. The most detailed experience with direct cortical stimulation has come from Penfield, himself, and is discussed further in the section below.

## Penfield’s Prediction

The synchronized parallel forebrain hypothesis is, in actuality, simply an elaboration of Penfield’s “centrencephalic system,” which he emphasized so often in his teaching. Penfield defined the centrencephalic system as “neurone circuits which must unite the two hemispheres within the [diencephalon]” (9); he described it as “a system of nerve fiber tracts and … gray matter within the [diencephalon] which plays the major role in the organization of the function of the two hemispheres” (9). He clearly imagined this system had special importance in integrating disparate neuronal circuits into a unified whole.

Penfield paid special attention to distinguish the parts of the neural apparatus, which were part of his “centrencephalic system” and those which were not. “Those parts and circuits … which may be shown to serve the purposes of inter-relationship of the cortical gray matter of the two hemispheres may be defined as the centrencephalic system and only those.” (9). Penfield’s distinction is completely consistent with the distinction above of the unsynchronized circuits (primary sensors and final effectors) from the synchronized circuits. He writes:

Specific sensory pathways from periphery to cortex, such as those of the visual system through the lateral geniculate bodies to calcarine cortex, do not form a part of the centrencephalic system, and neither does the somatic sensory path through the ventrolateral nucleus of the thalamus to the postcentral gyrus. But the further projections (which are still to be described clearly) from the various areas of sensory cortex into the central integrating area do form a part of the centrencephalic system. The neuronal pathways that emerge from unidentified centrally placed ganglia carrying volitional impulses to the precentral gyrus of each hemisphere do form a part of the centrencephalic system but the specific cortico-bulbo-spinal pathways which carry the impulses to the motor neurons of the bulb and cord do not. (9).

A point should be made here about the secondary sensory cortices. There is evidence from Penfield’s work that stimulation of second order sensory areas, such as the extra-calcarine cortex, also produces a sensation of flashing light (interestingly, in both visual fields as opposed to only the contralateral field) (9). This suggests that artificial electrical stimulation here is still capable of some degree of physiologic incorporation into the neural processing apparatus in at least a rudimentary way, despite the fact higher level and ongoing synchronized integration could not occur as it does under normal physiologic conditions in which higher order visual distinctions are made.

Interestingly, Penfield also wrote of “positive psychical responses which have been produced by electrical stimulation of the superior and lateral surfaces of the temporal lobes,” but noted that they “are clearly of a different physiological order from those produced by stimulation elsewhere in the brain” (9). I suspect these cases are best explained in the same fashion as the occasional stimulatory effect seen following stimulation of second order sensory cortices. Here, the artificial stimulation is still capable of some partial physiologic incorporation into the neural processing apparatus in at least a rudimentary way, although certainly not in the higher level and ongoing synchronized integration fashion that occurs under normal physiologic conditions. This should not come as a complete surprise given the occasional psychical symptoms that occur following the pathologic abnormal cortical discharge in patients with complex partial seizures. In Penfield’s words, “If ‘psychical states’ can be produced by epileptic discharge occurring in a hyper-irritable area of gray matter, why should they not be produced also by electrical stimulation?” (9).

Penfield noted that direct cortical stimulation of the *primary motor cortex* can produce both “interference” (an ablative effect, which results in the loss of the ability of the cortex to perform its normal motor functions) and a movement response (a stimulatory effect) (9). Penfield suggested: “When it does produce movement [a stimulatory effect], it is by virtue of conduction of impulses from cortex to ganglionic areas of the cerebrospinal axis.” (9). In this case, it is layer V neurons, which project directly to motor effectors that are being stimulated. The “interference” (or ablative) effect can be explained by interruption of the synchronized signals, which are normally processed outside of layer V of the primary motor cortex.

Penfield further notes, that simulation of the primary motor cortex can, on occasion, produce more complicated movements, which he felt were “activation of in-born reflexes of [the] brainstem” (9). He pointed out that “these movements are the same as those that the chronic decerebrate animal continues to make after the hemispheres have been removed, i.e., vocalization, swallowing, mastication, conjugate looking movement of the eyes … lifting of the head in response to sound, kicking and running … growling …. These … were reflex reactions in response to stimulus from without, differing from spinal reflexes only by being more complex” (9). All of these are examples of stimulation of final effectors in the motor system broadly defined by Swanson. Therefore all of these would be expected to be unsynchronized, and therefore produce a stimulatory effect by artificial high-frequency electrical stimulation. Penfield further anticipated the synchronized parallel forebrain hypothesis and the ablative effects of DBS by writing that under normal physiologic conditions “the pattern of the voluntary impulses that reach the precentral gyrus from the central zone of integration determines the nature of … voluntary movements. The rigid rhythm of the electrical current cannot imitate this pattern” (9).

## Conclusion

Further elaboration of Wilder Penfield’s classic centrencephalic system concept provides a possible answer to the current perplexing problem of the mechanism of action of DBS. Of more general relevance, however, if the synchronized parallel forebrain hypothesis is correct, then it represents a fundamental concept in the understanding of the functional organization of the forebrain. The forebrain provides the neural apparatus necessary for the temporal synchronization of multiple independent streams of processed information, which is necessary for parallel processing. Large-scale parallel processing provides us with the ability to produce high level abstract neural constructs and flexible combinations of voluntary movements to interact with ever changing environmental situations. Increasingly sophisticated parallel processing has emerged with the elaboration of the forebrain in higher vertebrates, culminating in our own species. On a practical level, artificial high-frequency electrical stimulation of tightly synchronized circuits results in loss of physiological (or pathophysiological) function of that circuit. This fact can be exploited to produce beneficial therapeutic effects in diseases of the human nervous system.

## Conflict of Interest Statement

The author declares that the research was conducted in the absence of any commercial or financial relationships that could be construed as a potential conflict of interest.
